# A generation at risk: a cross-sectional study on HIV/AIDS knowledge, exposure to mass media, and stigmatizing behaviors among young women aged 15–24 years in Ghana

**DOI:** 10.1080/16549716.2017.1331538

**Published:** 2017-06-16

**Authors:** Charity Konadu Asamoah, Benedict Oppong Asamoah, Anette Agardh

**Affiliations:** ^a^ International Master Programme in Public Health, Faculty of Medicine, Lund University, Malmö, Sweden; ^b^ Social Medicine and Global Health, Department of Clinical Sciences, Lund University, Malmö, Sweden

**Keywords:** Ghana, HIV/AIDS, youth, young women, stigma, discrimination, sexual reproductive health and rights

## Abstract

HIV/AIDS stigmatizing behaviors are a huge barrier to early detection and treatment of individuals with the AIDS virus. HIV/AIDS stigma and related consequences are debilitating, especially for vulnerable populations. This study sought to assess whether young women’s HIV/AIDS knowledge levels and exposure to mass media (television and radio) have an influence on their stigmatizing behaviors and role as agents of stigma towards individuals living with HIV and AIDS. The data used for this study originated from the Ghana Multiple Indicator Cluster Survey 2011. Binary and multiple (stepwise) logistic regression analyses were used to examine the associations between HIV/AIDS knowledge, frequency of exposure to mass media, and HIV/AIDS stigmatizing behaviors among young women aged 15–24 years in Ghana. Of the 3573 young women, 80% of 15–19-year-olds and 76% of 20–24-year-olds had at least one stigmatizing behavior towards persons living with HIV/AIDS (PLHA). Young women with increased knowledge regarding HIV/AIDS and frequent exposure to mass media (television and radio) had lesser tendency to stigmatize or act as agents of stigma towards PLHA (proportion with at least one stigmatizing behavior per subgroup – HIV/AIDS knowledge: those with highest knowledge score 579 [70.1%], those with lowest knowledge score 28 [90.3%]; mass media: those with daily exposure 562 [73.4%], those not exposed at all 249 [89.2%]). There was a graded negative ‘exposure–response’ association between the ranked variables: HIV/AIDS knowledge, mass media, and HIV/AIDS stigmatizing behaviors. The significant inverse association between HIV/AIDS knowledge, frequency of exposure to mass media, and HIV/AIDS stigmatizing behaviors persisted even after adjusting for all other covariates in the multiple logistic regression models. It is extremely important to increase HIV/AIDS-related knowledge and reduce stigma among young women in Ghana through targeted HIV/AIDS factual knowledge transfer. The use of mass media for communication of issues regarding HIV/AIDS, its mode of transmission, and associated stigma should be emphasized among women in Ghana.

## Background

Sub-Saharan Africa alone accounts for 71% of persons living with HIV and AIDS (PLHA). The pandemic has become a major public health issue in this region [1] with very serious socioeconomic and psychological effects on PLHA, their families, and their countries.

Young people are generally considered to be the most vulnerable group with regard to HIV/AIDS, as they become sexually active and engage in risky sexual behaviors including unprotected sexual intercourse [2,3]. The burden of HIV and AIDS among young people continues to be on the rise [4]. Young women (15–24 years) are at greater risk of being infected with HIV compared to their male counterparts. Although the prevalence of HIV and AIDS significantly dropped from 1.9% in 2013 to 1.6% in 2014 in Ghana [5], the proportion of infected women is higher than that of men, with cases among young women accounting for more than half of the prevalence in young people [5,6].

Stigma plays a huge role as a barrier in the prevention and control of HIV/AIDS [7–10]. Factors contributing to HIV and AIDS stigma and discrimination include lack of education and knowledge concerning the etiology of HIV and AIDS and its mode of transmission [11], misconceptions concerning the transmission, the life-threatening nature of the disease, and the epidemiology of the disease [12–16].

The stigma associated with the disease often excludes PLHA from seeking voluntary counseling and testing services, and accessing the highly effective antiviral drugs that exist [7–10,17]. A growing number of studies in the past decade have addressed the issue of HIV/AIDS-related stigma [18–20].

One such study conducted in South Africa in 2004 and 2008 concluded that individuals with higher knowledge scores on HIV/AIDS, previously tested for HIV, as well as those who knew a family member or friend who was infected with HIV or had died of AIDS had significantly lower stigma scores [19].

Among the Ghanaian population, voluntary counseling and testing for HIV are low, which in many studies is attributed to the stigma and discrimination associated with HIV [5,16,21]. This is very detrimental to individuals’ health and the health of the community [22].

Studies have shown that mass media (television, radio, etc.) is a powerful way of sending public health and health promotion messages to the population and particularly for sending culturally sensitive messages to young people [23,24]. However, no previous study has been conducted in young Ghanaian women concerning the relationship between exposure to mass media (television and radio), HIV/AIDS knowledge, and HIV/AIDS stigma. The aim of this study was thus to examine the extent to which knowledge of HIV/AIDS and its transmission and frequent exposure to mass media (television and radio) influenced stigmatizing behaviors among young Ghanaian women aged 15–24 years.

## Methods

### Study design, data collection, and study population

**Figure 1.**
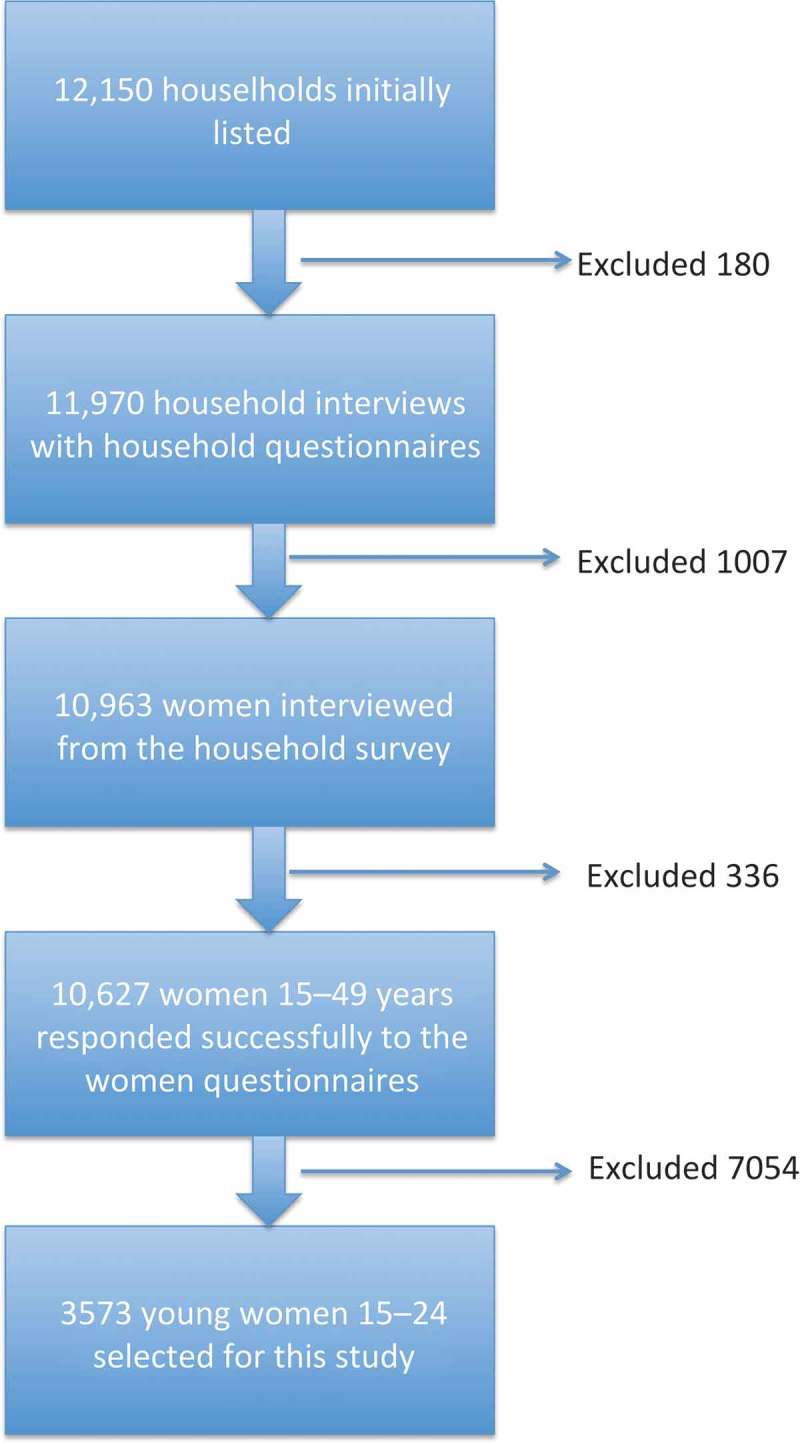
Flow chart of sample selection process for this study using the 2011 Ghana MICS.

This cross-sectional study used data that originated from the women’s questionnaire included in the Ghana Multiple Indicator Cluster Survey (MICS) 2011 [25]. The details of the data collection process have been outlined in Figure 1. This current study used a sub-sample of 3573 young women aged 15–24 years, i.e. the most vulnerable group affected and infected by HIV/AIDS as well as its associated stigma. The sample included respondents from all the 10 administrative regions of Ghana.

### Measures

#### Outcome

HIV/AIDS stigmatizing behavior was the outcome of interest for this study. This study used indicators of social distancing to assess HIV/AIDS stigma in the form of potential behaviors, thoughts, feelings, and discriminatory attitudes that express prejudice against PLHA. Detailed explanation of all the measures is provided in the Supplemental Data.

#### Main exposures

##### HIV/AIDS knowledge

The variable *HIV/AIDS knowledge* was created using responses to nine questions on HIV/AIDS knowledge. The total HIV/AIDS knowledge score ranged from 0 to 9 (‘no question answered correctly’ to ‘all questions correctly answered’) where a score of zero represented no HIV/AIDS knowledge and a score of nine represented the highest HIV/AIDS knowledge. HIV/AIDS knowledge was further regrouped into six homogeneous subgroups (0 = least to 5 = highest knowledge; 0 [0], 1–2 [1], 3 [2], 4 [3], 5–7 [4], and 8–9 [5]) based on an initial analysis using box plots.

##### Frequency of exposure to mass media

Frequency of exposure to television and radio was independently assessed by the questions: (a) *do you watch television?*and (b) *do you listen to radio?*The response options were (1) *almost every day*, (2) *at least once a week*, (3) *less than once a week,* and (4) *not at all.*

### Statistical analysis

The data were analyzed using Statistical Package for the Social Sciences version 22.0 (SPSS 22). Chi-squared tests and simple and multiple logistic regression were used for analyses. A *p*-value of 0.05 was chosen as the significance threshold.

## Results

A total of 3573 young women were included in the study of which 1899 (53.2%) were 15–19 years old and 1674 (46.8%) were 20–24 years. About 1 out of 4 (23.5%) had the highest HIV/AIDS knowledge score whereas 0.9% scored 0 (the lowest). About 78% of the participants reported at least one stigmatizing behavior towards PLHA. Stigmatizing behavior against male PLHA in the workplace was slightly higher (49.7%) compared with female PLHA (43.1%). Stigmatizing behavior against PLHA was more prevalent in the community (70.1%) than in the family (28.6%). Detailed sample characteristics are provided in Table S2 in the Supplemental Data.

Table 1 shows the proportions of different subgroups of young women who exhibited potential stigmatizing behaviors towards PLHA. Eighty percent of young women aged 15–19 years and 76% of those 20–24 years old had at least one stigmatizing behavior towards PLHA. About 70% of those with the highest HIV/AIDS knowledge score expressed at least one stigmatizing behavior towards PLHA compared with 90.3% among those with no HIV/AIDS knowledge. About 73% of participants who had daily access to mass media (television and radio) reported HIV/AIDS stigmatizing behaviors towards PLHA compared with 89.2% who reported not using mass media at all. Participants with secondary/higher education expressed lower stigmatizing behaviors towards PLHA (64.8%) compared to those with no education (89.2%).

**Table 1.** Prevalence (including *p*-values for square tests) of HIV/AIDS stigmatizing behaviors among different subgroups of young Ghanaian women aged 15–24 years in Ghana according to the Ghana MICS 2011.

At least one stigmatizing behavior				
	No N (%)	Yes N (%)	Total	*p*-values
**Age**				
15–19	372 (20)	1492 (80)	1864 (100)	.006
20–24	392 (23.8)	1257 (76.2)	1649 (100)	
**HIV knowledge score (6-point scale: 0 = lowest, 5 = highest)**				<0.001
.00	3 (9.7)	28 (90.3)	31 (100)	
1.00	22 (10.9)	180 (89.0)	202 (100)	
2.00	59 (11.4)	457 (88.6)	516 (100)	
3.00	170 (19.1)	721 (80.9)	891 (100)	
4.00	261 (25.0)	784 (75.0)	1045 (100)	
5.00	247 (29.9)	579 (70.1)	826 (100)	
**Mass media (total)**				<0.001
Not at all	30 (10.8)	249 (89.2)	279 (100)	
Occasionally	103 (16.6)	516 (83.4)	619 (100)	
Moderate	128 (21.5)	468 (78.5)	596 (100)	
Very frequent but not daily	300 (23.9)	954 (76.1)	1254 (100)	
Daily	204 (26.6)	562 (73.4)	766 (100)	
**Formal education**				<0.001
No education	26 (10.3)	226 (89.7)	252 (100)	
Basic education	418 (17.8)	1934 (82.8)	2352 (100)	
Secondary/higher	320 (35.2)	589 (64.8)	909 (100)	
**Wealth index**				<0.001
Poorest	51 (10.4)	441 (89.6)	492 (100)	
Poorer	114 (17.6)	535 (82.4)	649 (100)	
Poor	163 (21.1)	610 (78.9)	773 (100)	
Richer	177 (22.9)	597 (77.1)	774 (100)	
Richest	259 (31.4)	566 (68.6)	825 (100)	
**Literacy level**				<0.001
Cannot read at all	98 (11.6)	746 (88.4)	844 (100)	
Can read part of a sentence	68 (14.5)	400 (85.5)	468 (100)	
Can read a whole sentence	276 (21.6)	1004 (78.4)	1280 (100)	
**Ever been tested for AIDS virus**				<0.001
Yes	195 (29.0)	477 (71.0)	672 (100)	
No	488 (20.2)	1925 (79.8)	2413 (100)	
**Area of residence**				<0.001
Urban	502 (26.1)	1425 (73.9)	1927 (100)	
Rural	262 (16.5)	1325 (83.5)	1587 (100)	
**Region of residence**				<0.001
Western	38 (11.7)	288 (88.3)	326 (100)	
Central	77 (20.5)	298 (79.5)	375 (100)	
Greater Accra	159 (25.2)	473 (74.8)	632 (100)	
Volta	39 (14.3)	233 (85.7)	272 (100)	
Eastern	97 (24.7)	295 (75.3)	392 (100)	
Asante	178 (24.9)	538 (75.1)	716 (100)	
Brong Ahafo	89 (23.3)	293 (76.7)	382 (100)	
Northern	34 (17.3)	162 (82.7)	196 (100)	
Upper east	31 (24.0)	98 (76.0)	129 (100)	
Upper west	22 (23.9)	70 (76.1)	92 (100)	
**Ethnicity**				.001
Akan	389 (23.3)	1282 (76.7)	1671 (100)	
Ga Damgme	90 (29.4)	216 (70.6)	306 (100)	
Ewe	81 (17.3)	388 (82.7)	469 (100)	
Guan	24 (18.0)	109 (82.0)	133 (100)	
Gruma	15 (12.5)	105 (87.5)	120 (100)	
MoleDagbani	119 (20.6)	460 (79.4)	579 (100)	
Grusi	24 (17.6)	112 (82.4)	136 (100)	
Mande	9 (24.3)	28 (75.7)	37 (100)	
Non-Ghanaian	6 (15.4)	33 (84.6)	39 (100)	
Other ethnic	6 (26.1)	17 (73.9)	23 (100)	

Table 2 shows the results (crude odds ratios, 95% confidence intervals) of the bivariate analyses for the different categorical variables with respect to HIV/AIDS stigmatizing behaviors. The odds of having stigmatizing behaviors increased with decreasing level of HIV/AIDS knowledge. Young women with very low HIV knowledge had more than three times higher odds of having HIV/AIDS stigmatizing behavior. The odds of having stigmatizing behaviors increased with decreasing frequency of use of mass media. Participants with no access to mass media had three times higher odds of HIV/AIDS stigmatizing behavior compared to those with daily access to mass media (television and radio).

**Table 2.** Logistic regression analysis showing the association between age, HIV/AIDS knowledge, frequency of exposure to mass media, education, wealth, and HIV/AIDS stigmatizing behaviors among young women aged 15–24 years in Ghana using the Ghana MICS 2011.

	Crude odds ratio (95% CI)
**Age**	
15-19	1.25 (1.06 – 1.47)
20-24	Ref
**HIV/AIDS knowledge score (6 scale: 0-lowest, 5- highest)**	
00	4.10 (1.23 – 13.75)
1.00	3.43 (2.16 – 5.46)
2.00	3.28 (2.41 – 4.47)
3.00	1.81 (1.45 – 2.26)
4.00	1.28 (1.05 – 1.57)
5.00	Ref
**Mass Media (total)**	
Not at all	3.05 (2.02 – 4.61)
Occasionally	1.82 (1.39 – 2.37)
Moderate exposure	1.33 (1.03 – 1.71)
Very frequent but not daily	1.15 (0.94 – 1.42)
Daily	Ref
**Formal education**	
No education	4.74 (3.09 – 7.28)
Basic education	2.51 (2.11 – 2.98)
Secondary/higher education	Ref
**Wealth index**	
Poorest	3.98 (2.87–5.51)
Second	2.14 (1.67–2.75)
Middle	1.71 (1.37–2.15)
Fourth	1.54 (1.23–1.93)
Richest	Ref
**Literacy**	
Can not read at all	2.09 (1.63–2.68)
Can read part	1.62 (1.21–2.16)
Can read whole	Ref
**Ever been tested for the AIDS virus**	
No	1.62 (1.33–1.96)
Yes	Ref
Area of residence	
Urban	Ref
Rural	1.78 (1.51–2.11)
Region of Residence	
Western	2.54 (1.73–3.73)
Central	1.29 (0.95–1.76)
Greater Accra	Ref
Volta	2.00 (1.36–2.93)
Eastern	1.02 (0.79–1.30)
Asante	1.01 (0.79–1.30)
Brong Ahafo	1.12 (0.82–1.49)
Northern	1.61 (1.07–2.43)
Upper east	1.05 (0.68–1.64)
Upper west	1.08 (0.65–1.80)
**Ethnicity**	
Akan	Ref
Ga Damgme	0.96 (0.73–1.26)
Ewe	1.52 (1.16–1.99)
Guan	1.33 (0.87–2.05)
Gruma	3.11 (1.72–5.62)
Mole Dagbani	1.30 (1.03–1.65)
Grusi	1.14 (0.73–1.78)
Mande	1.19 (0.50–2.79)
Non Ghanaian	1.66 (0.78–3.54)
Other ethnic	0.86 (0.34–2.20)

Note: CI = confidence interval.

Table 3 presents multivariate stepwise analyses of the association between HIV/AIDS knowledge and HIV/AIDS stigmatizing behaviors. Generally, young women with lesser HIV/AIDS knowledge were still at higher risk of stigmatizing PLHA.

**Table 3.** Multiple logistic regressions including adjusted odds ratios with 95% confidence intervals showing the association between HIV/AIDS knowledge, age, area, wealth, and education with HIV/AIDS stigmatizing behaviors among young women aged 15–24 years in Ghana.

Variable	Model 1	Model 2	Model 3	Model 4	Model 5	Model 6
**HIV knowledge score (6-point scale)**						
.00	4.02 (1.20–13.49)	3.73 (1.10–12.63)	3.69 (1.09–12.54)	2.94 (0.86–10.02)	2.30 (0.67–7.90)	1.82 (0.53–6.27)
1.00	3.39 (2.13–5.39)	3.34 (2.09–5.33)	3.25 (2.03–5.21)	2.98 (1.86–4.78)	2.63 (1.63–4.23)	2.22 (1.37–3.60)
2.00	3.26 (2.40–4.44)	3.42 (2.50–4.68)	3.33 (2.43–4.57)	3.17 (2.31–4.36)	2.91 (2.11–4.01)	2.36 (1.70–3.28)
3.00	1.80 (1.44–2.25)	1.85 (1.47–2.32)	1.85 (1.47–2.33)	1.76 (1.40–2.22)	1.62 (1.28–2.05)	1.41 (1.11–1.79)
4.00	1.27 (1.03–1.55)	1.29 (1.05–1.59)	1.30 (1.06–1.61)	1.26 (1.02–1.56)	1.23 (1.00–1.52)	1.15 (0.93– 1.43)
5.00	Ref	Ref	Ref	Ref	Ref	Ref
**Age**						
15–19	1.24 (1.05–1.46)	1.23 (1.04–1.45)	1.22 (1.04–1.45)	1.22 (1.03–1.44)	1.17 (0.98–1.38)	1.07 (0.90–1.28)
20–24	Ref	Ref	Ref	Ref	Ref	Ref
**Area of residence**						
Rural				1.61 (1.34–1.93)	1.20 (0.97–1.49)	1.14 (0.92–1.42)
Urban				Ref	Ref	Ref
**Wealth index**						
Poorest					3.56 (2.30–5.52)	2.84 (1.82–4.44)
Second					1.81 (1.33–2.46)	1.53 (1.12–2.09)
Middle					1.57 (1.21–2.03)	1.35 (1.04–1.76)
Fourth					1.49 (1.18–1.89)	1.40 (1.10–1.77)
Richest					Ref	Ref
**Formal education**						
No education						2.64 (1.65–4.24)
Basic education						1.83 (1.50–2.24)
Secondary +						Ref

Notes: Model 1: adjusted for age; Model 2: adjusted for age and region; Model 3: adjusted for age, region, and ethnicity; Model 4: Adjusted for age, region, ethnicity, and area (rural/urban); Model 5: adjusted for age, region, ethnicity, area (rural/urban), and wealth; Model 6: adjusted for age, region, ethnicity, area (rural/urban), wealth, and education.

Table 4 shows the results of the multivariate stepwise analyses of the association between HIV/AIDS stigmatizing behavior and mass media exposure.

**Table 4.** Multiple logistic regressions including adjusted odds ratios with 95% confidence intervals showing the association between frequency of exposure to mass media, age, area, and education with HIV/AIDS stigmatizing behaviors among young women aged 15–24 years in Ghana.

	Model 1	Model 2	Model 3	Model 4	Model 5
**Mass media (total)**					
Not at all	2.94 (1.95–4.46)	3.06 (1.99–4.73)	2.98 (1.92–4.63)	2.53 (1.62–3.94)	1.85 (1.17–2.91)
Occasionally	1.76 (1.34–2.30)	1.72 (1.30–2.27)	1.68 (1.27–2.22)	1.50 (1.14–1.99)	1.21 (0.90–1.61)
Moderate exposure	1.30 (1.01–1.68)	1.25 (0.96–1.62)	1.21 (0.93–1.58)	1.09 (0.84–1.43)	0.90 (0.69–1.19)
Very frequent but not daily	1.12 (0.91–1.34)	1.14 (0.92–1.40)	1.13 (0.91–1.39)	1.08 (0.87–1.34)	0.97 (0.78–1.20)
Daily	Ref	Ref	Ref	Ref	Ref
**Age**					
15–19	1.19 (1.01–1.40)	1.17 (0.99–1.38)	1.17 (0.99–1.39)	1.17 (0.99–1.38)	1.06 (0.89–1.26)
20–24	Ref	Ref	Ref	Ref	Ref
**Area of residence**					
Rural				1.61 (1.34–1.93)	1.40 (1.15–1.68)
Urban				Ref	Ref
**Formal education**					
No education					3.67 (2.30–5.85)
Basic education					2.23 (1.84–2.71)
Secondary +					Ref

Notes: Model 1: adjusted for age; Model 2: adjusted for age and region; Model 3: adjusted for age, region, and ethnicity; Model 4: adjusted for age, region, ethnicity, and area (rural/urban); Model 5: adjusted for age, region, ethnicity, area (rural/urban), and education.

The final model adjusted for age, region, ethnicity, area of residence, and education. ‘Not at all’ users of mass media were twice as likely to stigmatize PLHA compared to the reference group. The models that included mass media exposure were not adjusted for wealth status since calculation of the wealth index encompassed ownership of household assets such as television and radio.

## Discussion

The study revealed a statistically significant association between increased HIV/AIDS knowledge and decreased HIV/AIDS stigmatizing behaviors among young women (15–24 years) in Ghana. A statistically significant association between more frequent exposure to mass media (radio and television) and decreased stigmatizing behaviors towards PLHA was also found in the same population.

The findings that HIV/AIDS stigmatizing behaviors were reduced among young women with the highest HIV/AIDS knowledge are consistent with those from previous studies [16,19,26], showing that lack of factual knowledge about the mode of transmission of the AIDS virus and the corresponding myths associated with AIDS transmission contribute to the stigmatizing behaviors and discriminatory attitudes towards PLHA. Therefore, it is extremely important to promote efforts to increase young women’s HIV/AIDS-related knowledge. It is also critically important to create multiple avenues for young women to receive information on sexual reproductive health and rights issues such as HIV/AIDS knowledge, especially in settings where the opportunity for young women to be enrolled in formal education may be limited.

The present study also found that frequent exposure to mass media (TV and radio) decreases young women’s stigmatizing behaviors towards PLHA. This is consistent with previous studies that reported mass media as one of the important sources of HIV/AIDS information in terms of prevention and decreasing stigmatizing behaviors [23,27,28]. Therefore, collaboration between the media companies and the various governmental and non-governmental organizations in Ghana to intensify HIV/AIDS messages to youth and to the entire Ghanaian population is highly recommended. The results indicate the need for restructuring and re-evaluating those programs already existing in the Ghanaian mass media, such as the ‘Heart to Heart’ HIV campaign and ‘Protect your Goal’ campaign [29]. These efforts are mostly organized by PLHA to provide HIV/AIDS messages, themes, or stories of HIV/AIDS on prime-time and daytime entertainment such as *telenovelas* (television novels) to maximize their positive effects among the youth. Specifically designed programs could be initiated for targeting both the agents of stigmatization and the stigmatized. According to Xiao and colleagues [28] and Singhal and Rogers [30], mass media, especially television, is a powerful medium for communicating sensitive health messages as well as being able to effectively change health behaviors in individuals with low literacy levels who otherwise might find it difficult to process such messages. Health messages should be available and reinforced in already existing informal gatherings such as youth centers, churches, and community and market gatherings to help young women acquire the right HIV/AIDS knowledge for self-protection as well as acceptance of peers who are infected with the virus. Level of education may not only be a confounder but could be in the same causal chain, where exposure to media mediates the effect of education on discriminatory attitudes/behavior. Thus, raising the general level of education could be an important strategy for reducing stigma and discrimination against HIV-positive individuals.

The study also found that the majority of young women (70.1%) were unwilling to buy fresh vegetables from a person that they knew had the AIDS virus, but in contrast, most (71.4%) of them were willing to care for a family member infected with the AIDS virus. This finding is consistent with previous studies [16,19]. Therefore, young women could benefit from education that emphasizes detailed and correct knowledge about the AIDS disease and how to deal with stigma and discrimination.

Finally, there was a statistically significant association between increased formal education, being wealthy, and urban residence, and decreased HIV/AIDS stigmatizing behaviors. This requires appropriate intervention from the government to create opportunities aimed at bridging the socioeconomic gap among the youth, including young women. There should be periodic evaluation of the approaches used in countering HIV/AIDS stigmatizing behaviors among young women and tailored HIV/AIDS information and messages to specific cultures and groups. Further research is needed to examine the nature of the association between the use of social media or other mass media, including the Internet, as a tool for HIV/AIDS knowledge transmission and reduction of stigmatizing behaviors.

### Methodological considerations

The comprehensive nature of the information available in the data-set and the representativeness of the entire 10 administrative regions of Ghana are major strengths for this study. Therefore, the results may be generalized to all Ghanaian young women who are 15–24 years of age. Additionally, this is the first study of its kind in Ghana to examine the impact of media exposure on HIV/AIDS stigmatizing behaviors. The graded negative association between the ranked HIV/AIDS knowledge, mass media exposure, and HIV/AIDS stigmatizing behaviors lends further support to the validity of the findings. Causality cannot be inferred between the covariates and HIV/AIDS stigmatizing behaviors due to the cross-sectional nature of the data. Also, the study used secondary data that were self-reported, which can be subject to bias. On the one hand, using an aggregated ‘series of questions’ to define stigmatizing behaviors could help minimize underreporting of the stigmatizing behaviors due to the socially sensitive nature of the issue. On the other, young women with higher HIV/AIDS knowledge could have underreported their potential stigmatizing behaviors, due to a bias towards social desirability.

## Conclusion

The present study suggests an urgent need to reduce HIV/AIDS stigmatizing behaviors among young women in Ghana through comprehensive sexuality education using dynamic approaches. It is extremely important to promote programs that increase HIV/AIDS-related knowledge among young women, especially among those with low wealth and/or low education. The use of mass media for communication or knowledge transfer concerning issues related to HIV/AIDS, its mode of transmission, and associated stigma should be emphasized among women in Ghana.

## Supplementary Material

Supplemental data

## Paper context

Previous studies in the general population have shown an association between low HIV/AIDS knowledge and stigma and discrimination towards persons living with HIV/AIDS (PLHA). In recent years, the Ghana AIDS Commission, UNAIDS, and PLHA have developed health messages concerning HIV/AIDS transmission, prevention, and associated stigma for various mass media campaigns, using PLHA as spokespersons. This is the first study that has been conducted in young Ghanaian women concerning the relationship between exposure to mass media (television and radio), HIV/AIDS knowledge, and HIV/AIDS stigma.
