# Supplementary material for: A generation at risk: a cross-sectional study on HIV/AIDS knowledge, exposure to mass media, and stigmatizing behaviors among young women aged 15–24 years in Ghana
Source: Glob Health Action. 2017 Jun 16;10(1):1331538. doi: 10.1080/16549716.2017.1331538 (PMC5496072; doi:10.1080/16549716.2017.1331538)
Supplement: Supplemental data [file ZGHA_A_1331538_SM0214.docx]

**Measures**

**Outcome**

HIV/AIDS stigmatizing behavior was the outcome of interest for this study. This study used indicators of social distancing to assess HIV/AIDS stigma in the form of potential behaviors, thoughts, feelings and discriminatory attitudes that express prejudice against people living with HIV or AIDS. The main questions in the Ghana MICS used to identify HIV/AIDS stigmatizing behaviors were as follows: *1) In your opinion, if a female teacher has the AIDS virus but is not sick, should she be allowed to continue teaching in school?* This question was used in the current study as a proxy for stigma in the workplace against females living with HIV*. 2) In your opinion, if a male teacher has the AIDS virus but is not sick, should he be allowed to continue teaching in school?* This question was used as a proxy for stigma in the workplace against males living with HIV*. 3) Would you buy fresh vegetables from a shopkeeper or vendor if you knew that this person had the AIDS virus?* This question was used as a proxy for stigma against PLHA in the community*. 4) If a member of your family become sick with AIDS, would you be willing to care for her or him in your own house?* This question was used as a proxy for stigma against PLHA in the family*.* The responses were dichotomized as “yes” and “no”. “No” indicates potential stigmatizing behaviors in the respective settings/environment towards individuals infected with HIV/AIDS whereas “yes,” indicates no potential stigmatizing behavior. Responses to the four stigma questions described above were scored as 1 = “stigmatizing behavior” and 0 = “no stigmatizing behavior”. The four individual stigma scores were then summed up to create an aggregate stigmatizing behavior ordinal scale ranging from zero to four in increasing magnitude of stigmatizing behavior, where zero indicates “no” to the entire set of stigma questions (no stigmatizing behavior) and four indicates “yes” to all the questions (most stigmatizing behaviors). The outcome “stigmatizing behavior” was used both as an ordinal categorical variable (with five categories, 0 – 4) and further dichotomized as 0 (*No stigmatizing behavior*) and 1 (*1– 4, one or more stigmatizing behaviors*).

**Main exposures:**

*HIV/AIDS knowledge*

The variable *HIV/AIDS knowledge* was created using responses to the following nine questions: *1) Can people reduce their chance of getting the AIDS virus by having just one uninfected sex partner who has no other sex partners? 2) Can people get the AIDS virus because of witchcraft or other supernatural means? 3) Can people reduce their chance of getting the AIDS virus by using condom every time they have sex? 4) Can people get the AIDS virus from mosquito bites? 5) Can people get the AIDS virus by sharing food with a person who has the AIDS virus? 6) Is it possible for a healthy-looking person to have the AIDS virus? Can the virus that causes AIDS be transmitted from a mother to her baby: 7) during pregnancy 8) during delivery 9) by breastfeeding?* Each of these questions generated a dichotomized response “yes or no”. All the correct answers were scored (one point for each correct answer) and summed together. The total HIV/AIDS knowledge score ranged from 0 to 9 (‘no *question answered correctly’* to ‘*all questions correctly answered’*) where a score of zero (0) represented no HIV/AIDS knowledge and a score of nine represented the highest HIV/AIDS knowledge. For simplicity and to enable sufficient sample size in the respective groups for further analysis, crude analysis of the association between HIV knowledge score (10 scale, 0-9) and the outcome (HIV/AIDS stigmatizing behavior) was done using box plots and simple logistic regression to identify homogenous subgroups within the 10 scale HIV/AIDS knowledge categories. Following the initial analysis, HIV/AIDS knowledge was further regrouped into six homogeneous subgroups (0 = least to 5 = highest knowledge) (0 [0], 1 – 2 [1], 3 [2], 4 [3], 5 – 7 [4] and 8 – 9 [5])

*Frequency of exposure to mass media*

Frequency of exposure to television and radio was independently assessed by the questions: *a) do you watch television? and b) do you listen to radio? The response options were 1) almost every day, 2) at least once a week, 3) less than once a week 4) not at all. These were recoded for simplicity as* ‘*1’ representing* ‘*not at all’ and 4 as almost everyday.* Responses to both questions (television and radio) were summed and yielded a total score ranging from two to eight (two = not at all and eight = daily). Five scales were created out of the 8 scales by combining homogeneous groups together (8 [5], 6 – 7[4], 5[3], 3 – 4[2], 2[1]) after initial analysis with the outcome using boxplots and crude logistic regressions.

**Other Covariates**

*Age* was dichotomized as 15 – 19 years and 20 – 24 years as these two groups often differ characteristically in matters relating to sexual reproductive health and rights.

*Education* was assessed by the question: “what is the highest level of school you have attended?” The response options were: preschool=0, primary=1, middle/ JSS/ JHS=2, secondary/SSS/SHS/Tech/Vocational =3, higher=4 and other specify=5. The responses were recoded into three categories as: 1) No education (or only preschool) corresponding to never having attended any formal educational system; 2) Basic education corresponding to some level of education up to Primary/JSS, equivalent to 6 – 9 years of formal education; and 3) Secondary/higher education corresponding to at least 12 or more years of education, including secondary or tertiary education.

*Wealth* status was assessed by the wealth index quintile (poorest, second, middle, fourth, and richest). The MICS uses the World Bank’s Gross National Income per capita as a baseline to develop the wealth index quintiles, which refer to the general wealth of the woman’s household.

*Ever tested for the AIDS virus* was assessed by the question “I don’t want to know the results but have you ever tested to see if you have the AIDS virus”? The response options were “yes” and “no”.

*Literacy* level was assessed by the question “can you read part of a sentence”? The response options were: cannot read at all, able to read only part of a sentence, and able to read a whole sentence.

*Region* had the response options; Western, Central, Greater Accra, Volta, Eastern, Asante, Brong Ahafo, northern, upper east, upper west corresponding to the 10 administrative regions of residence in Ghana.

*Area of residence* was coded as urban and rural.

*Ethnicity* was assessed by the question “what is your ethnic group”. The response options were: Akan, Ga/Gamgme, Ewe, Guan, Gruma, Mole Dagbani, Grusi, Mande, non-Ghanaian and other ethnic groups.
